# Supplementary material for: Skin Cutibacterium acnes Mediates Fermentation to Suppress the Calcium Phosphate-Induced Itching: A Butyric Acid Derivative with Potential for Uremic Pruritus
Source: J Clin Med. 2020 Jan 22;9(2):312. doi: 10.3390/jcm9020312 (PMC7074307; doi:10.3390/jcm9020312)
Supplement: Supplementary file 1 [file jcm-09-00312-s001.pdf]

Article

Skin *Cutibacterium acnes* Mediates Fermentation to Suppress the Calcium Phosphate-Induced Itching: A Butyric Acid Derivative with Potential for Uremic Pruritus

Sunita Keshari [1], Yanhan Wang [2], Deron Raymond Herr [3], Sung-Min Wang [4], Wu-Chang Yang [5], Tsung-Hsien Chuang [6], Chien-Lung Chen [5,*] and Chun-Ming Huang[4,*]

[1] Department of Life Sciences, National Central University, Taoyuan, 32001, Taiwan; Ph.D. Student; sunitakeshari827@gmail.com
[2] Department of Dermatology, University of California, San Diego, California 92093, USA; Research Scientist; yaw015@ucsd.edu
[3] Department of Pharmacology, National University of Singapore, Singapore 117600; Assistant Professor; phcdrh@nus.edu.sg
[4] Department of Biomedical Sciences and Engineering, National Central University, Taoyuan 32001, Taiwan; Ph.D. Student; sss810621@yahoo.com.tw
[5] Division of Nephrology, Landseed International Hospital, Taoyuan, 32001, Taiwan; Physician; wuchang.yang2@gmail.com
[6] Immunology Research Center, National Health Research Institutes, Zhunan, Miaoli County 350, Taiwan; Investigator; thchuang@nhri.edu.tw
* Correspondence: chencl4922@gmail.com (C.-L.C.); Tel.: 03-4941234-2911; Fax: 03-2831281
chunming@ncu.edu.tw (C.-M.H.); Tel.: +886-3-422-7151; Fax: +886-3-425-3427

Received: 11 December 2019; Accepted: 20 January 2020; Published: date

1. Supplementary Information

1.1. ELISA

KERTr cells were treated with acetic acid, propionic acid, butyric acid or BA-NH-NH-BA in the presence or absence of CaP and then lysed with RIPA buffer supplemented with an EDTA-free protease inhibitor cocktail (Sigma, Burlington, MA, USA). Skin was collected from mice that were injected with CaP and treated with topical application of *C. acnes* plus glucose, butyric acid or BA-NH-NH-BA. Total protein was extracted by T-PER™ Tissue Protein Extraction Reagent for measurements of IL-6 by an ELISA assay kit (R&D Systems, Minneapolis, MN, USA).

1.2. Western blotting.

DRGs were isolated 2 h after treatment by CaP injection and topical application of *C. acnes* plus glucose, butyric acid or BA-NH-NH-BA. DRG lysates were subjected to 10% SDS-PAGE, transferred to a PVDF membrane (Millipore) and blocked with 5% (*w*/*v*) non-fat milk before incubation overnight with primary antibodies to p-ERK 1/2 (1:1,000, Cell Signaling, Danvers, MA, USA), GAPDH (1:1,000) or β-actin (1:1,000, Cell Signaling). This was followed by treatment for 1 h with horseradish peroxidase (HRP)-conjugated secondary antibody (goat anti-rabbit or anti-mouse (1:5,000). Protein bands were detected with a chemiluminescent detection reagent and Omega Lum™ C Imaging System (Gel Company, San Francisco, CA, USA). Densitometric analysis of the protein bands was conducted using Image J software.

1.3. Immunostaining

KERTr cells (0.3×10^7 cells/mL) were grown on 8-well chamber, treated with PBS, 4 mM butyric acid or BA-NH-NH-BA for 24 h, fixed in 4% formaldehyde (Sigma, Burlington, MA, USA) for 10 min, permeabilized with 0.3% Triton X-100 for 10 min and blocked in 5% BSA for 1 h prior to incubating with anti-acetyl lysine antibody (Abcam, Cambridge, UK) followed by Alexa Fluor®568 secondary antibody. Nuclei were counterstained with DAPI (4',6-diamidino-2-phenylindole) (1:5,000) and mounted with Lab Vision™ PermaFluor™ Aqueous Mounting Medium. All images were taken with an Olympus BX41 microscope and shown are representative of at least in triplicate.

1.4. Minimum bactericidal concentration (MBC)

C. acnes (10^5 CFU/mL) was incubated with 4 mM butyric acid or PBS on a 96-well microtiter plate for 24 h. After incubation, *C. acnes* was diluted $1:10^0$–$1:10^5$ with PBS. The number of *C. acnes* bacteria was determined by spotting the dilution (10 μL) on a reinforced clostridial medium (RCM) agar plate for the counting of CFUs.

1.5. 16S rRNA sequencing and Phylogenetic analysis

The 16S rRNA gene of *C. acnes* isolated from human skin was sequenced using Applied Biosystems 3730xl DNA Analyzer, USA [1]. The sequences were aligned with 16S rRNA sequences from various subtypes of *C. acnes* including ATCC 6919 (GenBank accession no. AB042288.1), KPA171202 (GenBank accession no. AE017283.1), NCTC 10390 (GenBank accession no. AY642044.1) and Asn12 (GenBank accession no. DQ672259.1). The phylogenetic relationships were determined by using the Data Analysis in Molecular Biology and Evolution (DAMBE) software (https://www.ebi.ac.uk/Tools/msa/muscle/). Multiple sequence alignments (MSA) were performed by using the CLUSTAL W algorithm [2,3] and exported into the DAMBE program. The phylogenetic tree was constructed by the maximum-parsimony method.

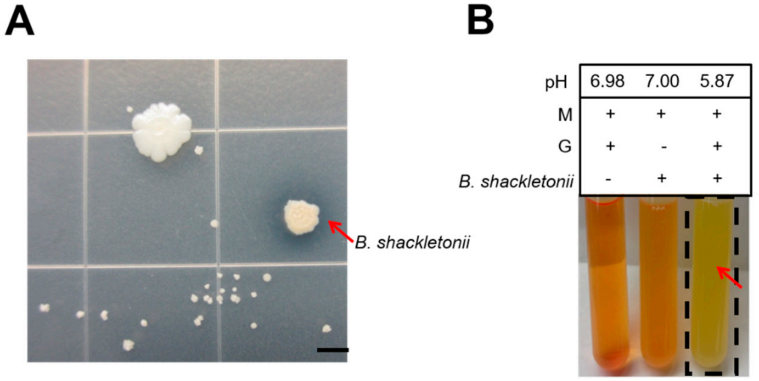

Figure S1. CaP- solubilizing property and glucose fermentation of *B. shackletonii.* (**A**) *B. shackletonii* solubilizes CaP indicated by a clear zone (red arrow) in a Pikovskaya's agar plate supplemented with glucose. Scale bar = 1 cm. (**B**) *B. shackletonii* was cultured in phenol red-containing rich media (M) in the presence of 2% glucose (G). Media with glucose or bacteria alone were used as controls. The yellowish media (red arrow) and a drop in pH value (5.87 vs 6.98 and 7.00 in controls) indicated the glucose fermentation of *B. shackletonii.*

A

GTGCATGCGCTGCTACACATGCAGTCGACGGAAGGCCCTGCTTTTGTGGGGTG
CTCGAGTGGCGAACGGGTGAGTAACACGTGAGTAACCTGCCCTTGACTTTGGG
ATAACTTCAGGAAACTGGGGCTAATACCGGATAGGAGCTCCTGCTGCATGGTG
GGGGTTGGAAAGTTTCGGCGGTTGGGGATGGACTCGCGGCTTATCACCTTGTT
GGTGGGGTAGTGGCTTACCAAGGCTTTGACGGGTAGCCGGCCTGACAAGGTG
ACCGGCCACATTGGGACTGAGATACGGCCCAAACTCCTACAGGAGGCAGCAAT
GGGGAATATTGCACAATGGGCGGAAGCCTGATGCACCAACGCCGCGTGCGGG
ATGACGGCCTTCGGGTTGTAAACCGCTTTCGCCTGTGACGAAGCGTGAATGAC
GGTAATGGGTAAAGAAGCACCGGCTAACTACGTGCCAGCAGCCGCGGTAATCT
AACTACGTGCCAGCAACCGCGGTAATA

B

CNAGNGGAGCTATAATGCAGTCGTGCGGACCTTTTAAAGCTTGCTTTTAAAAGG
TTAGCGGCGGACGGGTGAGTAACACGTGGGCAACCTGCCTGTAAGATCGGGA
TAACGCCGGGAAACCGGGGCTAATACCGGATAGTTTTTCCTCCGCATGGAGG
AAAAAGGAAAGACGGCTTTTGCTGTCACTTACAGATGGGCCCGCGGCGCATTA
GCTAGTTGGTGGGGTAACGGCTCACCAAGGCAACGATGCGTAGCCGACCTGA
GAGGGTGATCGGCCACATTGGGACTGAGACACGGCCCAAACTCCTACGGGAG
GCAGCAGTAGGGAATCTTCCGCAATGGACGAAAGTCTGACGGAGCAACGCCG
CGTGAGTGAAGAAGGCCTTCGGGTCGTAAAACTCTGTTGCCGGGGAAGAACAA
GTGCCGTTCGAACAGGGCGGCGCCTTGACGGTACCCGGCCAGAAAGCCACGG
CTAACTACGTAACAGCAG

Figure S2. 16S rRNA sequence of human isolated *C. acnes* (A) and *B. shackletonii* (B).

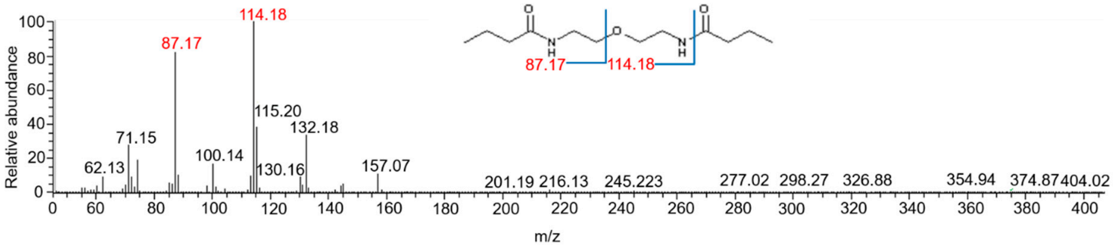

Figure S3. Mass spectrum of BA-NH-NH-BA. BA-NH-NH-BA ($C_{12}H_{24}N_2O_3$) (0.5 mL in H_2O) was analyzed by GC-MS. Resulting peaks at 87.17 and 114.18 m/z correspond to fragmentation of BA-NH-NH-BA in mass spectra analysis.

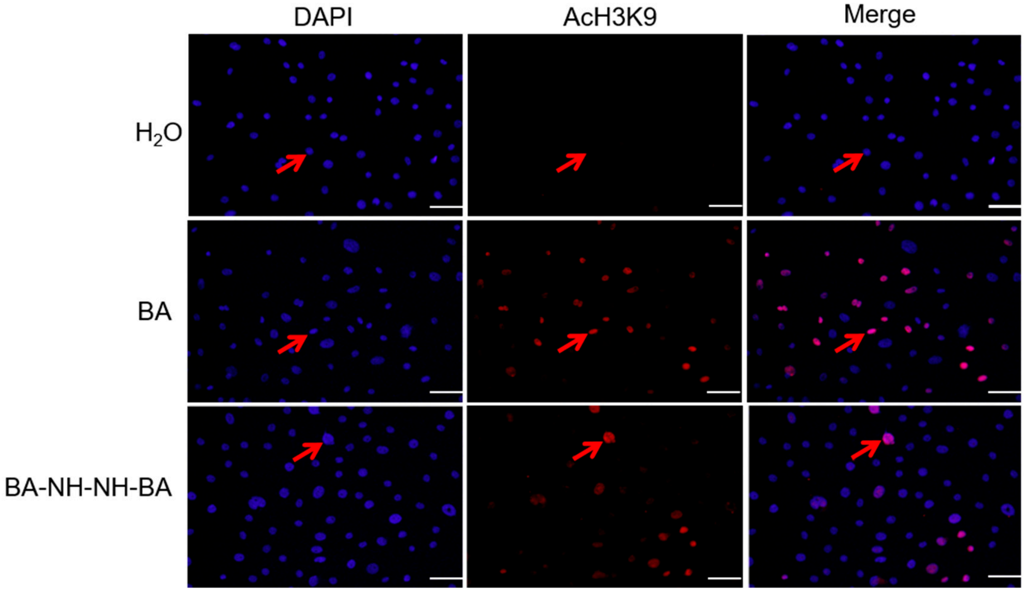

Figure S4. AcH3K9 in KERTr cells treated with BA or BA-BH-NH-BA. KERTr cells (0.3×10^7 cells/mL) were treated with 4 mM BA or BA-NH-NH-BA in H_2O for 24 h. Cells were treated with H_2O as a control. The presence of AcH3K9 was detected by immunostaining with an antibody to AcH3K9 (red dots; red arrows) and counter-staining with 4′,6-diamidino-2-phenylindole (DAPI) (nuclei; blue dots). Bars = 100 μm.

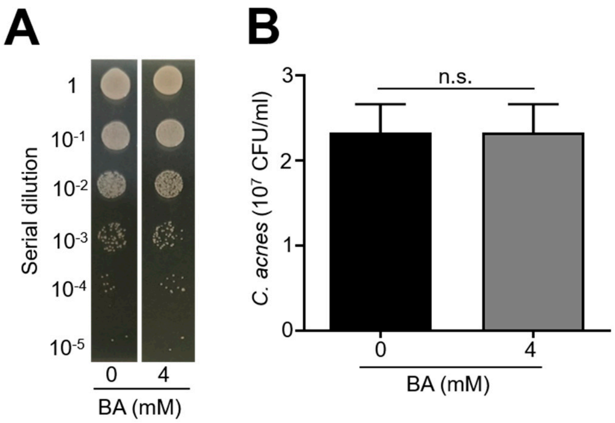

Figure S5. The effect of butyric acid on the growth of *C. acnes*. *C. acnes* (10^5 CFU/mL) was cultured with butyric acid (BA) (4 mM in PBS) for 24 h. After serial dilutions ($1{:}10^0$–$1{:}10^5$) of bacterial cultures, (**A**) the colonies of *C. acnes* were detected on agar plates and the number (10^7 CFU/mL) of bacterial colonies was determined (**B**). Data are the mean ± SE of three individual experiments. n.s. = non-significant.

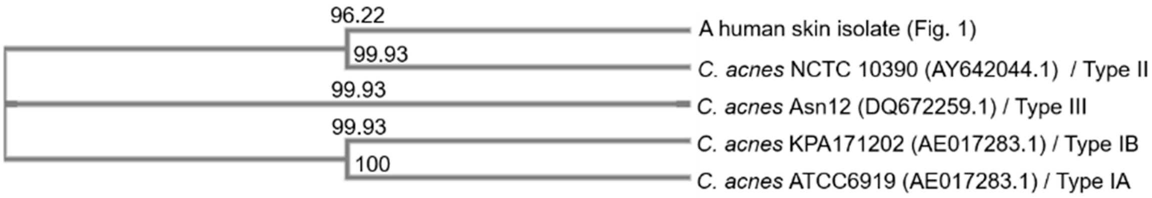

Figure S6. Phylogenetic tree of newly isolated *C. acnes* and known subtypes of *C. acnes* based on the 16s RNA gene sequences. Multiple sequence alignment was performed on the strain of *C. acnes* newly isolated from human skin relative to published sequences of *C. acnes* subtypes; type IA (ATCC 6919), type IB (KPA171202), type II (NCTC 10390) and type III (Asn12). The resulting phylogenetic tree of the human skin isolated *C. acnes* was rooted with the other *C. acnes* subtypes.

References

1. Grice, E.A.; Kong, H.H.; Renaud, G.; Young, A.C.; Program, N.C.S.; Bouffard, G.G.; Blakesley, R.W.; Wolfsberg, T.G.; Turner, M.L.; Segre, J.A. A diversity profile of the human skin microbiota. *Genome Res* **2008**, *18*, 1043-1050, doi:10.1101/gr.075549.107.
2. Caporaso, J.G.; Lauber, C.L.; Walters, W.A.; Berg-Lyons, D.; Lozupone, C.A.; Turnbaugh, P.J.; Fierer, N.; Knight, R. Global patterns of 16S rRNA diversity at a depth of millions of sequences per sample. *Proc Natl Acad Sci U S A* **2011**, *108 Suppl 1*, 4516-4522, doi:10.1073/pnas.1000080107.
3. DeSantis, T.Z.; Hugenholtz, P.; Larsen, N.; Rojas, M.; Brodie, E.L.; Keller, K.; Huber, T.; Dalevi, D.; Hu, P.; Andersen, G.L. Greengenes, a chimera-checked 16S rRNA gene database and workbench compatible with ARB. *Appl Environ Microbiol* **2006**, *72*, 5069-5072, doi:10.1128/AEM.03006-05.
